# Trends in Adiposity and Food Insecurity Among US Adults

**DOI:** 10.1001/jamanetworkopen.2020.12767

**Published:** 2020-08-07

**Authors:** Candice A. Myers, Emily F. Mire, Peter T. Katzmarzyk

**Affiliations:** 1 Pennington Biomedical Research Center, Baton Rouge, Louisiana

## Abstract

**Importance:**

Food insecurity is a pervasive public health issue in the US that is associated with greater body weight.

**Objective:**

To examine national trends in food insecurity among US adults from 1999 to 2016 according to surrogate measures of adiposity (body mass index [BMI] and waist circumference [WC]).

**Design, Setting, and Participants:**

This cross-sectional study analyzed nationally representative data obtained from nine 2-year cycles (1999 to 2000 through 2015 to 2016) of the National Health and Nutrition Examination Survey in the US. The sample comprised adult survey participants aged 20 years or older. Data analyses were performed from July 1, 2019, to March 31, 2020.

**Main Outcomes and Measures:**

The primary outcome was food insecurity. Data on BMI (calculated as weight in kilograms divided by height in meters squared) were categorized as follows: normal weight (BMI, <25), overweight (BMI, 25-29.9), and obese (BMI, ≥30). Data on WC were categorized as follows: less high risk (men: ≤102 cm; women: ≤88 cm) or high risk (men: >102 cm; women: >88 cm). Food insecurity prevalence by adiposity was further analyzed using key demographic characteristics, including sex and race/ethnicity (non-Hispanic White, non-Hispanic Black, Hispanic, and Other).

**Results:**

Among the 46 145 adults in the final sample, the mean (SD) age was 46.9 (0.2) years. Of this sample, 23 957 were women (52.0%; 95% CI, 51.6%-52.5%) and 20 825 were non-Hispanic White adults (68.8%; 95% CI, 66.6%-71.0%). The estimated prevalence of food insecurity was 18.2% (95% CI, 15.3%-21.2%) in the 2015 to 2016 cycle, a statistically significant change from 8.7% (95% CI, 7.3%-10.2%) in the 1999 to 2000 cycle. Among all adiposity categories, food insecurity prevalence significantly increased from the 1999 to 2000 cycle to the 2015 to 2016 cycle. The prevalence of food insecurity was highest in adults with obesity (22.6%; 95% CI, 19.5%-25.8%; *P* for trend <.001). For both men and women, food insecurity prevalence significantly increased from the 1999 to 2000 cycle (men: 8.8% [95% CI, 6.9%-10.6%]; women: 8.7% [95% CI, 7.0%-10.5%]) to the 2015 to 2016 cycle (men: 17.2% [95% CI, 14.1%-20.2%]; women: 19.2% [95% CI, 16.2%-22.2%]; *P* for trends <.001). For non-Hispanic White and non-Hispanic Black adults, food insecurity prevalence increased from the 1999 to 2000 cycle (non-Hispanic White: 6.0% [95% CI, 4.0%-8.0%]; non-Hispanic Black: 12.4% [95% CI, 9.6%-15.2%]) to the 2015 to 2016 cycle (non-Hispanic White: 13.0% [95% CI, 9.8%-16.3%]; non-Hispanic Black: 29.1% [95% CI, 24.2%-34.0%]; *P* for trends <.001). For Hispanic adults, food insecurity prevalence significantly increased in those with obesity (1999-2000: 19.1% [95% CI, 12.0%-26.1%]; 2015-2016: 37.6% [95% CI, 33.7%-41.5%]; *P* for trend ≤.001) and in both less-high-risk WC (1999-2000: 19.6% [95% CI, 12.8%-26.4%]; 2015-2016: 32.5% [95% CI, 27.3%-37.7%]; *P* for trend = .02) and high-risk WC categories (1999-2000: 19.3% [95% CI, 12.7%-25.9%]; 2015-2016: 36.7% [95% CI, 31.7%-41.7%]; *P* for trend <.001).

**Conclusions and Relevance:**

In this cross-sectional study, the estimated prevalence of food insecurity appeared to increase from 1999 to 2016 and across all levels of adiposity. These results suggest the need for multidisciplinary approaches to address the association between food insecurity and obesity in the US.

## Key Points

**Question:**

What were the national trends in food insecurity by measures of adiposity among US adults from 1999 through 2016?

**Findings:**

In this cross-sectional study of the National Health and Nutrition Examination Survey cycles, which included 46 145 adults, the estimated prevalence of food insecurity increased from approximately 9% in the 1999 to 2000 cycle to 18% in the 2015 to 2016 cycle. Women with greater adiposity had higher odds of food insecurity compared with those with less adiposity.

**Meaning:**

Findings of this study suggest that food insecurity is an increasingly prevalent public health issue in the US and has a significant association with greater adiposity.

## Teaser

This cross-sectional study examines the prevalence of food insecurity in the context of obesity and other sociodemographic factors among adults aged 20 years or older.

## Introduction

Food insecurity is a core indicator of nutritional status and “exists whenever the availability of nutritionally adequate and safe foods or the ability to acquire acceptable foods in socially acceptable ways is limited or uncertain.”^1^^(p1560)^ In the US, 11.1% of households reported being food insecure in 2018.^2^ Food insecurity is a public health concern, given its association with multiple chronic diseases,^3,4^ including type 2 diabetes,^5,6,7^ and poor cardiovascular health.^7,8,9,10,11^ A meta-analysis concluded that food insecurity was statistically significantly associated with obesity, with this association being most robust in women from high-income countries.^12^ Given the association between food insecurity and poor health, health care spending is greater in adults with food insecurity compared with those with food security.^13^

Although the US Department of Agriculture monitors food insecurity among US households,^2^ it is useful to analyze national trends in food insecurity by relevant health metrics.^7,14^ Because food insecurity is associated with greater body weight, especially among women, temporal trend analyses according to level of adiposity are needed to better prioritize efforts and resources aimed at reducing health disparities associated with food insecurity. To achieve this goal, we examined the national trends in food insecurity among US adults overall and by adiposity. Specifically, we used 2 surrogate measures of adiposity, body mass index (BMI) and waist circumference (WC), because these measurements are suitable proxies for body fat and are recommended for assessing adiposity in clinical settings.^15,16^ Trends in food insecurity by adiposity were further examined by key demographic characteristics, including sex and race/ethnicity.

## Methods

### Data Source and Study Population

For this cross-sectional study, we obtained nationally representative data from the National Health and Nutrition Examination Survey (NHANES). The NHANES assesses the health and nutritional status of adults and children in the US using data collected through interviews and physical examinations.^17^ The survey has been continuous since 1999 and involves a nationally representative sample of approximately 5000 people per year.^17^ In this study, we used nine 2-year cycles of NHANES data from 1999 to 2000 through 2015 to 2016, which was the most recent cycle in which data on food security were available. The sample was restricted to adults 20 years or older. Participants in the NHANES provide written informed consent, and study procedures are approved by the National Center for Health Statistics Research Ethics Review Board. The Pennington Biomedical Research Center Institutional Review Board exempted the present study because of its use of deidentified data. We followed the Strengthening the Reporting of Observational Studies in Epidemiology (STROBE) reporting guideline.^18^

### Assessment of Food Insecurity and Adiposity

Food security status was measured through the US Household Food Security Survey Module (developed by the US Department of Agriculture), which comprises 18 items that assess the “conditions and behaviors that characterize … difficulty meeting basic food needs” during the previous 12 months.^2^^(p2)^^19^ Three or more affirmative responses in this module indicate food insecurity.

Physical examinations included height, weight, and WC measurements, with BMI calculated as weight in kilograms divided by height in meters squared. Participant BMIs were categorized as follows: underweight (<18.5), normal weight (18.5-24.9), overweight (25-29.9), or obese (≥30).^15^ For analyses, underweight was included in the normal weight category. Waist circumference was measured in centimeters and dichotomized as less high risk (men: ≤102 cm; women: ≤88 cm) or high risk (men: >102 cm; women: >88 cm).^15^

### Assessment of Sociodemographic Characteristics

Sociodemographic characteristics included sex, race/ethnicity, age, educational level, poverty-to-income ratio (PIR), and marital status, which were self-reported in standardized questionnaires. Race/ethnicity groups were non-Hispanic White, non-Hispanic Black, Hispanic, and Other (which included race/ethnicity other than non-Hispanic White, non-Hispanic Black, and Hispanic, such as multiracial). Race/ethnicity was assessed according to evidence of greater prevalence of obesity and food insecurity among minority populations.^20,21^ Educational level was dichotomized as high school or less or some college or more. The PIR was dichotomized as income of 130% of or less than the federal poverty level (FPL) or income greater than 130% of the FPL. Households with an income 130% of or less than the FPL qualify for the Supplemental Nutrition Assistance Program, which addresses food insecurity by providing monthly benefits to qualifying low-income households.^2^ Marital status was dichotomized as married or not married.

### Statistical Analysis

We computed food insecurity prevalence (crude) and 95% CIs within each 2-year survey cycle by BMI and WC categories, sex, and race/ethnicity. Linear trends (crude) in food insecurity by BMI and WC categories according to sex and race/ethnicity are presented through line graphs. We evaluated linear trends (crude) using weighted logistic regression across survey cycles to calculate *P* values for trends. Survey cycle was used as a continuous variable in the regression models. Weighted logistic regression models, including appropriate sociodemographic covariates (age, educational level, PIR, and marital status), were used to estimate the odds of food insecurity by BMI and WC categories, sex, and race/ethnicity. Furthermore, we included interaction terms between BMI or WC and sex and race/ethnicity in weighted logistic regression models to examine effect modification. Then, we analyzed stratified models as indicated by statistically significant interaction terms. Participants with missing data were excluded from analyses.

The complex survey design for the NHANES includes oversampling, stratification, and clustering, and each record is assigned a weight to account for several features of the survey, including unequal sampling probability and nonresponse.^22,23^ All analyses were appropriately conducted with survey analysis procedures to account for the complex survey design.

We conducted analyses with SAS, version 9.4 (SAS Institute Inc). A 2-sided *P* ≤ .05 was used to determine significance. Data analyses were performed from July 1, 2019, to March 31, 2020.

## Results

The final sample size for this study was 46 145 individuals, with a mean (SD) age of 46.9 (0.2) years. Of this sample, 23 957 were women (52.0%; 95% CI, 51.6%-52.5%), 22 188 were men (48.0%; 95% CI, 47.5%-48.4%), and 20 825 were non-Hispanic White adults (68.8%; 95% CI, 66.6%-71.0%). This sample represented 210 628 042 noninstitutionalized US adults age 20 years or older. Table 1 presents the weighted sample characteristics by food security status for all 9 cycles (1999-2016) of the NHANES. eTable 1 in the Supplement presents this information for the most recent cycle (2015-2016). Within the overall sample, 8118 adults (13.0%; 95% CI, 12.3% to 13.6%) reported being food insecure. Statistically significant differences between adults with food security vs those with food insecurity were seen for all descriptive characteristics. For example, the food insecure group had a greater proportion of adults with obesity than the food secure group (41.5% [95% CI, 39.9%-43.0%] vs 33.5% [95% CI, 32.7%-34.4%]; *P* < .001). Similarly, the food insecure group vs the food secure group had a greater proportion of adults with a high-risk WC (53.7% [95% CI, 52.1%-55.3%] vs 50.7% [95% CI, 49.6%-51.8%]; *P* = .001).

**Table 1.** Sample Size and Weighted Sample Characteristics by Food Security Status Among US Adults, 1999-2016^a^

Characteristic	% (95% CI)		*P* value^b^
	Food secure group	Food insecure group	
Unweighted sample, No.	38 027	8118	NA
Weighted sample, No.^c^	183 280 726	27 347 316	NA
BMI			<.001
Normal weight: <25	32.3 (31.4-33.2)	29.0 (27.3-30.7)	
Overweight: 25-29.9	34.2 (33.5-34.9)	29.5 (28.0-30.9)	
Obese: ≥30	33.5 (32.7-34.4)	41.5 (39.9-43.0)	
WC, cm			.001
<High risk	49.3 (48.2-50.4)	46.3 (44.7-47.9)	
High risk	50.7 (49.6-51.8)	53.7 (52.1-55.3)	
Sex			.01
Women	51.8 (51.4-52.2)	53.5 (52.1-54.8)	
Men	48.2 (47.8-48.6)	46.5 (45.2-47.9)	
Race/ethnicity			<.001
Non-Hispanic White	72.1 (70.0-74.1)	46.8 (43.1-50.5)	
Non-Hispanic Black	10.2 (9.0-11.3)	18.6 (16.4-20.7)	
Hispanic	11.2 (9.8-12.7)	28.6 (25.4-31.7)	
Other^d^	6.6 (5.9-7.2)	6.1 (5.2-7.0)	
Age, y			<.001
20-39	35.7 (34.7-36.8)	49.7 (47.8-51.6)	
40-64	45.1 (44.2-45.9)	41.8 (40.2-43.4)	
≥65	19.2 (18.5-19.9)	8.5 (7.6-9.4)	
Educational level			<.001
≤High school	38.8 (37.4-40.3)	62.9 (61.1-64.8)	
≥Some college	61.2 (59.7-62.6)	37.1 (35.2-38.9)	
Poverty-to-income ratio (family)			<.001
≤130% FPL	21.7 (20.6-22.8)	61.1 (59.0-63.2)	
>130% FPL	78.3 (77.2-79.4)	38.9 (36.8-41.1)	
Marital status			<.001
Married	58.8 (57.7-59.9)	39.0 (37.1-40.9)	
Not married	41.2 (40.1-42.3)	61.0 (59.1-62.9)	

Abbreviations: BMI, body mass index (calculated as weight in kilograms divided by height in meters squared); FPL, federal poverty level; NA, not applicable; WC, waist circumference.

^a^ Data from the National Health and Nutrition Examination Survey.^17^ Data are presented as percentage (95% CI) unless indicated otherwise.

^b^ Calculated with χ^2^ tests to determine statistically significant differences between food security status groups.

^c^ Data were weighted to be nationally representative.

^d^ Other race/ethnicity included groups other than non-Hispanic White, non-Hispanic Black, and Hispanic, such as multiracial.

Table 2 presents crude weighted trends in food insecurity among adults by BMI, WC, sex, and race/ethnicity. The estimated prevalence of food insecurity was 18.2% (95% CI, 15.3%-21.2%) in the 2015 to 2016 cycle, a statistically significant change from the prevalence in the 1999 to 2000 cycle (8.7% [95% CI, 7.3%-10.2%]; *P* for trend <.001). Among all BMI and WC categories, food insecurity significantly increased from the 1999 to 2000 cycle through the 2015 to 2016 cycle. Across BMI categories in 2015 to 2016, food insecurity was highest in adults with obesity (22.6%; 95% CI, 19.5%-25.8%; *P* for trend <.001). Food insecurity was greater in adults with high-risk WC compared with adults with less high-risk WC (19.8% [95% CI, 16.8%-22.8%] vs 16.2% [95% CI, 12.8%-19.5%]; *P* for trends <.001). Both men and women experienced significant increases in food insecurity from the 1999 to 2000 cycle (men: 8.8% [95% CI, 6.9%-10.6%]; women: 8.7% [95% CI, 7.0%-10.5%]) to the 2015 to 2016 cycle (men: 17.2% [95% CI, 14.1%-20.2%]; women: 19.2% [95% CI, 16.2%-22.2%]; *P* for trends <.001). Among non-Hispanic White and non-Hispanic Black adults, food insecurity significantly increased from the 1999 to 2000 cycle (non-Hispanic White: 6.0% [95% CI, 4.0%-8.0%]; non-Hispanic Black: 12.4% [95% CI, 9.6%-15.2%]) to the 2015 to 2016 cycle (non-Hispanic White: 13.0% [95% CI, 9.8%-16.3%]; non-Hispanic Black: 29.1% [95% CI, 24.2%-34.0%]; *P* for trends <.001). Hispanic adults also saw significant increases, from 19.5% (95% CI, 13.6%-25.4%) in the 1999 to 2000 cycle to 35.0% (95% CI, 31.0%-38.9%) in the 2015 to 2016 cycle (*P* for trend <.001).

**Table 2.** Crude Weighted Trends in Food Insecurity Among US Adults, 1999-2016^a^^,^^b^

Variable	% (95% CI)									*P* for trend^c^
	1999-2000	2001-2002	2003-2004	2005-2006	2007-2008	2009-2010	2011-2012	2013-2014	2015-2016	
Overall	8.7 (7.3-10.2)	10.8 (8.8-12.8)	11.3 (9.9-12.8)	9.7 (8.1-11.2)	11.6 (9.6-13.6)	13.9 (12.1-15.7)	15.9 (12.9-18.8)	15.2 (13.0-17.5)	18.2 (15.3-21.2)	<.001
BMI										
Normal weight: <25	8.7 (6.3-11.1)	10.9 (7.7-14.0)	10.5 (8.1-12.9)	9.9 (7.2-12.6)	11.1 (8.7-13.5)	12.5 (10.7-14.3)	14.0 (9.9-18.1)	14.1 (10.4-17.7)	15.2 (11.5-18.8)	<.001
Overweight: 25-29.9	7.3 (5.1-9.5)	9.7 (7.3 - 12.0)	9.9 (7.0-12.7)	9.0 (7.2-10.9)	10.4 (8.4-12.4)	13.2 (10.8-15.6)	13.7 (10.2-17.2)	12.7 (9.8-15.5)	15.6 (11.7-19.4)	<.001
Obese: ≥30	10.4 (8.8-12.1)	11.5 (8.5-14.4)	13.7 (12.0-15.4)	9.9 (7.5-12.3)	13.3 (10.4-16.1)	15.7 (12.9-18.6)	19.7 (16.2-23.1)	18.4 (15.8-21.0)	22.6 (19.5-25.8)	<.001
WC										
<High risk	8.7 (6.1-11.3)	11.2 (8.8-13.7)	10.6 (8.8-12.4)	10.0 (8.0-12.0)	11.9 (9.8-14.1)	13.5 (11.9-15.2)	14.7 (11.1-18.4)	13.8 (10.9-16.6)	16.2 (12.8-19.5)	<.001
High risk	8.8 (7.7-9.9)	10.02 (8.0-12.4)	12.1 (10.1-14.0)	9.3 (7.4-11.2)	11.2 (9.0-13.4)	14.3 (11.9-16.7)	16.9 (13.8-20.0)	16.5 (14.2-18.8)	19.8 (16.8-22.8)	<.001
Sex										
Women	8.7 (7.0-10.5)	11.1 (8.7-13.3)	12.0 (10.5-13.5)	9.6 (7.7-11.5)	11.8 (9.6-14.0)	14.5 (12.5-16.6)	15.8 (12.8-18.7)	15.9 (13.1-18.6)	19.2 (16.2-22.2)	<.001
Men	8.8 (6.9-10.6)	10.4 (8.4-12.5)	10.6 (8.8– 12.4)	9.8 (8.1-11.4)	11.3 (9.4-13.2)	13.3 (11.5-15.1)	16.0 (12.5-19.5)	14.6 (12.3-16.9)	17.2 (14.1-20.2)	<.001
Race/ethnicity										
Non-Hispanic White	6.0 (4.0-8.0)	7.3 (4.9-9.6)	7.7 (5.7-9.7)	5.8 (4.4-7.2)	8.2 (6.0-10.3)	8.4 (6.6-10.3)	11.5 (8.5-14.4)	11.4 (9.3-13.6)	13.0 (9.8-16.3)	<.001
Non-Hispanic Black	12.4 (9.6-15.2)	16.6 (12.4-20.7)	18.5 (15.5-21.4)	17.6 (14.2-21.0)	17.5 (13.3-21.6)	28.1 (22.7-33.6)	26.2 (22.4-30.1)	23.2 (20.1-26.3)	29.1 (24.2-34.0)	<.001
Hispanic	19.5 (13.6-25.4)	26.3 (21.0-31.7)	29.1 (21.5-36.6)	23.3 (18.8-27.7)	23.9 (20.0-27.9)	31.1 (26.9-35.2)	28.8 (22.0-35.7)	28.0 (22.6-33.5)	35.0 (31.0-38.9)	<.001
Other	8.2 (0.8-15.6)	8.2 (0.6-15.9)	7.4 (2.8-12.0)	16.1 (9.4-22.8)	12.9 (6.8-19.0)	11.3 (6.5-16.1)	14.4 (9.4-19.4)	11.7 (5.8-17.7)	13.9 (10.0-17.8)	.08

Abbreviations: BMI, body mass index (calculated as weight in kilograms divided by height in meters squared); WC, waist circumference.

^a^ Data from the National Health and Nutrition Examination Survey.^17^ Data are presented as percentage (95% CI) for each survey cycle.

^b^ Data were weighted to be nationally representative.

^c^ The estimated β (95% CI) and *P* for trend were calculated using logistic regression that included the National Health and Nutrition Examination Survey 2-year cycle as a continuous variable.

**Figure 1.**
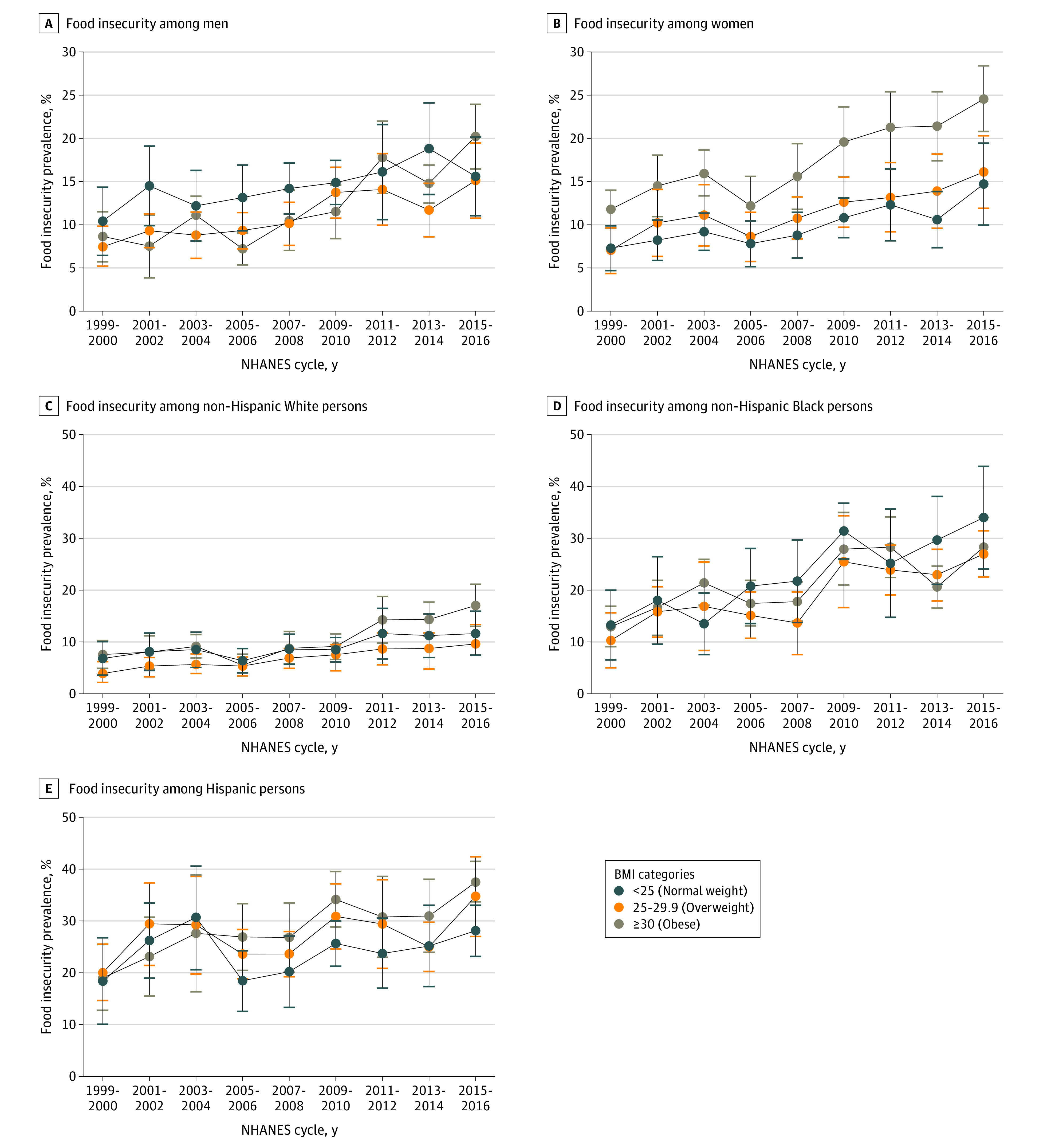
Crude Weighted Trends in Body Mass Index (BMI) by Sex and Race/Ethnicity Data from 1999 to 2016 National Health and Nutrition Examination Survey (NHANES).^17^ Data were weighted to be nationally representative. Error bars indicate 95% CI.

Figure 1 as well as eFigure 1 and eTable 2 in the Supplement show the crude weighted trends in food insecurity prevalence across categories of BMI and WC stratified by sex. For both men and women, food insecurity significantly increased in all BMI and WC categories from the 1999 to 2000 cycle through the 2015 to 2016 cycle. For men, food insecurity in 1999 to 2000 was highest in those with normal weight (10.4%; 95% CI, 6.5%-14.4%) compared with those with overweight (7.5%; 95% CI, 5.2%-9.8%) or obesity (8.6%; 95% CI, 5.7%-11.5%). However, in 2015 to 2016, food insecurity was highest in those with obesity (20.2%; 95% CI, 16.5%-24.0%; *P* for trend <.001) and was similar in those with overweight (15.1%; 95% CI, 10.8%-19.5%; *P* for trend <.001) or normal weight (15.6%; 95% CI, 11.3%-20.4%; *P* for trend = .008). In addition, among men, food insecurity was greater in those with high-risk WC (18.1%; 95% CI, 14.9%-21.3%; *P* for trend <.001) compared with those with less-high-risk WC (16.4%, 95% CI, 12.9%-20.0%; *P* for trend <.001) in 2015 to 2016. Among women in 1999 to 2000, food insecurity was greater in those with obesity (11.8%; 95% CI, 9.6%-14.0%) compared with those with overweight (7.0%; 95% CI, 4.4%-9.7%) or normal weight (7.3%; 95% CI, 4.7%-9.9%). In 2015 to 2016, food insecurity was greater in women with obesity (24.6%; 95% CI, 20.8%-28.4%; *P* for trend <.001) compared with women with overweight (16.1%; 95% CI, 11.9%-20.3%; *P* for trend <.001) or normal weight (14.7%; 95% CI, 9.9%-19.4%; *P* for trend <.001). In addition, for women, food insecurity was greater in those in the high-risk WC category (20.9%; 95% CI, 17.5%-24.4%; *P* for trend <.001) compared with those in the less-high-risk WC category (15.8%; 95% CI, 11.6%-20.0%; *P* for trend <.001) in 2015 to 2016.

Figure 1 as well as eFigure 1 and eTable 3 in the Supplement show the crude weighted trends in food insecurity by BMI and WC for each race/ethnicity group, except for the Other category because of insufficient sample size. For non-Hispanic White and non-Hispanic Black adults, food insecurity prevalence increased in all BMI and WC categories from the 1999 to 2000 cycle through the 2015 to 2016 cycle (*P* ≤ .01 for trends). Patterns in food insecurity by BMI and WC categories differed in these 2 racial/ethnic groups. Among non-Hispanic White adults in 2015 to 2016, food insecurity was greater in those with obesity (17.1%; 95% CI, 13.0%-21.1%) compared with those with overweight (9.6%; 95% CI, 5.8%-13.4%) and normal weight (11.7%; 95% CI, 7.4%-15.9). In addition, food insecurity was greater in non-Hispanic White adults with high-risk WC (14.7%; 95% CI, 11.0%-18.4%) compared with those with less-high-risk WC (10.6%; 95% CI, 7.5%-13.8%). For non-Hispanic Black adults in 2015 to 2016, food insecurity was greatest in those with normal weight (34.0%; 95% CI, 24.1%-43.9%) compared with those with overweight (27.0%; 95% CI, 22.6%-31.5%) and obesity (28.3%; 95% CI, 22.6%-34.1%). Furthermore, food insecurity was greater in non-Hispanic Black adults with less-high-risk WC (31.0%; 95% CI, 24.3%-37.6%) compared with those with high-risk WC (27.5%; 95% CI, 21.9%-33.2%). For Hispanic adults, food insecurity statistically significantly increased in those with obesity (1999-2000: 19.1% [95% CI, 12.0%-26.1%]; 2015-2016: 37.6% [95% CI, 33.7%-41.5%]; *P* for trend ≤.001) but not in those with normal weight (1999-2000: 18.4% [95% CI, 9.1%-27.7%]; 2015-2016: 28.1% [95% CI, 23.1%-33.0%]; *P* for trend = .34) or overweight (1999-2000: 20.1% [95% CI, 13.8%-26.3%]; 2015-2016: 34.7% [95% CI, 27.0%-42.4%]; *P* for trend = .06). Food insecurity prevalence also significantly increased in Hispanic adults with less-high-risk WC (1999-2000: 19.6% [95% CI, 12.8%-26.4%]; 2015-2016: 32.5% [95% CI, 27.3%-37.7%]; *P* for trend = .02) and those with high-risk WC (1999-2000: 19.3% [95% CI, 12.7%-25.9%]; 2015-2016: 36.7% [95% CI, 31.7%-41.7%]; *P* for trend <.001). In the most recent cycle of data (2015-2016), food insecurity prevalence was greater in Hispanic adults with greater adiposity, including those with obesity and high-risk WC, compared with those with less adiposity, including those with normal weight or overweight and less-high-risk WC.

Table 3 shows the results from weighted logistic regression models for estimating the odds of food insecurity by BMI, WC, sex, race/ethnicity, and sociodemographic characteristics. In the fully adjusted model (model 2), the odds of food insecurity were greater for adults with obesity (odds ratio [OR], 1.23; 95% CI, 1.08-1.39) than for those with normal weight (reference group). Compared with adults with less-high-risk WC (reference group), those with high-risk WC also had higher odds of food insecurity (OR, 1.13; 95% CI, 1.02-1.25). Men did not have higher odds of food insecurity (OR, 0.98; 95% CI, 0.92-1.05) compared with women (reference group). Both non-Hispanic Black adults (OR, 1.66; 95% CI, 1.48-1.86) and Hispanic adults (OR, 2.03; 95% CI, 1.78-2.31) had higher odds of food insecurity compared with non-Hispanic White adults (reference group). Adults with some college or more education had lower odds of food insecurity (OR, 0.53; 95% CI, 0.48-0.57) compared with adults with high school or less education (reference group). Compared with adults with a PIR that was 130% of or less than the FPL (reference group), adults with a PIR greater than 130% of the FPL had lower odds of food insecurity (OR, 0.25; 95% CI, 0.23-0.28). Adults who were not married had greater odds of food insecurity (OR, 1.47; 95% CI, 1.32-1.64) compared with those who were married (reference group).

**Table 3.** Weighted Logistic Regression Models of Food Insecurity, Adjusted for Adiposity, Sex, Race/Ethnicity, and Sociodemographic Characteristics, 1999-2016^a^

Variable	Odds ratio (95% CI)	
	Model 1^b^	Model 2^c^
BMI		
Normal weight: <25	1 [Reference]	1 [Reference]
Overweight: 25-29.9	0.89 (0.80-0.99)	0.95 (0.85-1.06)
Obese: ≥30	1.24 (1.10-1.38)	1.23 (1.08-1.39)
WC		
<High risk	1 [Reference]	1 [Reference]
High risk	1.00 (0.91-1.09)	1.13 (1.02-1.25)
Sex		
Women	1 [Reference]	1 [Reference]
Men	1.05 (0.99-1.12)	0.98 (0.92-1.05)
Race/ethnicity		
Non-Hispanic White	1 [Reference]	1 [Reference]
Non-Hispanic Black	2.74 (2.45-3.08)	1.66 (1.48-1.86)
Hispanic	3.91 (3.44-4.45)	2.03 (1.78-2.31)
Other	1.45 (1.22-1.73)	1.11 (0.92-1.33)
Age	NA	0.98 (0.98-0.98)
Educational level		
≤High school	NA	1 [Reference]
≥Some college	NA	0.53 (0.48-0.57)
Poverty-to-income ratio (family)		
≤130% FPL	NA	1 [Reference]
>130% FPL	NA	0.25 (0.23-0.28)
Marital status		
Married	NA	1 [Reference]
Not married	NA	1.47 (1.32-1.64)
Survey year		
1999-2000	NA	1 [Reference]
2001-2002	NA	1.37 (1.02-1.82)
2003-2004	NA	1.55 (1.28-1.87)
2005-2006	NA	1.42 (1.16-1.73)
2007-2008	NA	1.52 (1.21-1.91)
2009-2010	NA	1.89 (1.53-2.34)
2011-2012	NA	2.23 (1.79-2.78)
2013-2014	NA	2.13 (1.73-2.64)
2015-2016	NA	3.00 (2.45-3.66)

Abbreviations: BMI, body mass index (calculated as weight in kilograms divided by height in meters squared); FPL, federal poverty level; NA, not applicable; WC, waist circumference.

^a^ Data were weighted to be nationally representative. Participants with missing data were excluded from analyses.

^b^ Adjusted for adiposity, sex, and race/ethnicity.

^c^ Fully adjusted.

**Figure 2.**
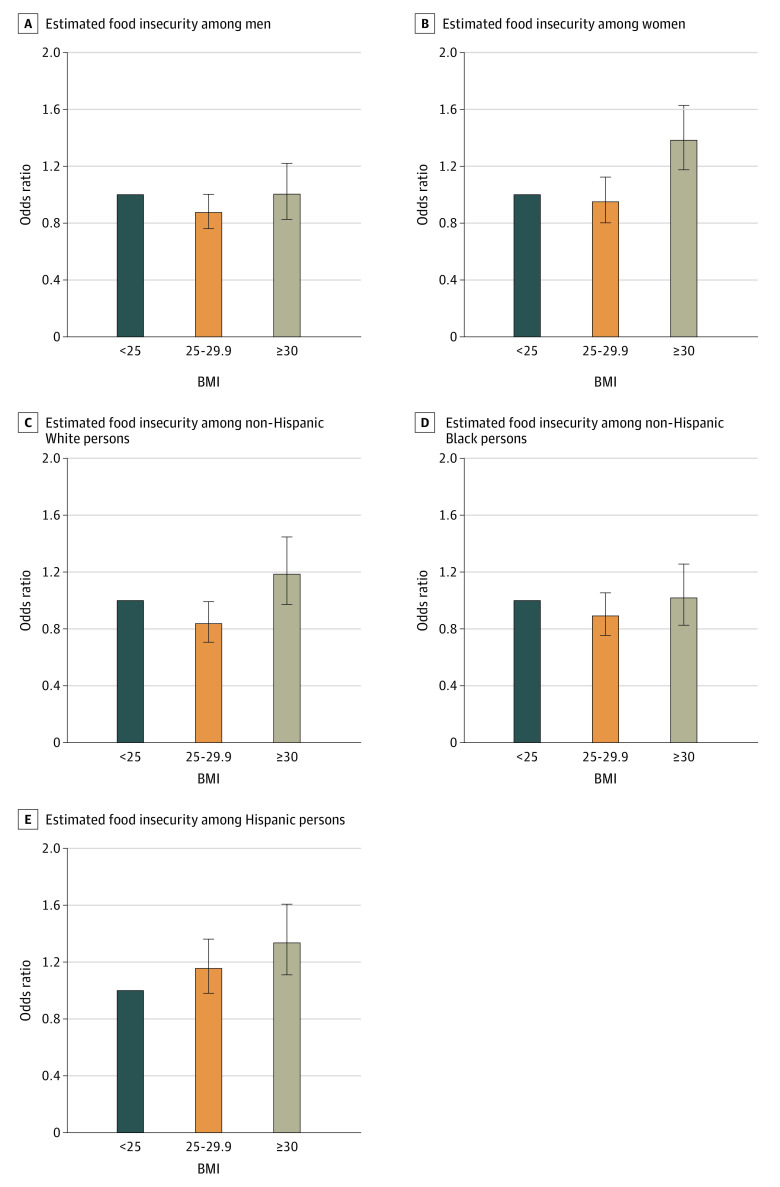
Adjusted Odds (95% CI) of Food Insecurity Among Adults in the US Data from 1999 to 2016 National Health and Nutrition Examination Survey.^17^ Data were weighted to be nationally representative. Body mass index (BMI [calculated as weight in kilograms divided by height in meters squared]) less than 25 is the reference category for the odds ratios presented; therefore, there are no error bars for this category. Error bars indicate the 95% CIs.

Given the statistically significant interactions between sex and adiposity, including both BMI (*F*_2,122_ = 3.51; *P* = .03) and WC (*F*_1,123_ = 4.25; *P* = .04), sex-stratified models were used for estimating food insecurity (Figure 2; eFigure 2 in the Supplement). The results indicated that women with obesity had greater odds of being food insecure (OR, 1.38; 95% CI, 1.18-1.63) compared with women with normal weight. In addition, women with high-risk WC had higher odds of food insecurity (OR, 1.32; 95% CI, 1.14-1.53) compared with those with less-high-risk WC. For men, the odds of food insecurity associated with BMI or WC were not significant. Race/ethnicity-stratified models were also estimated because of a significant interaction between BMI and race/ethnicity that identified food insecurity (*F*_6,118_ = 2.54; *P* = .02). Although the interaction between WC and race/ethnicity was not significant (*F*_3,121_ = 1.29; *P* = .28), given the established correlation between BMI and WC^24^ as well as that found in the present sample (*r* = 0.90; *P* < .001), we assessed race/ethnicity-stratified models for both adiposity measures (Figure 2). Non-Hispanic White adults with high-risk WC had higher odds of food insecurity (OR, 1.25; 95% CI, 1.06-1.48) compared with those with less-high-risk WC. For non-Hispanic Black adults, the odds of food insecurity associated with BMI or WC were not significant. Hispanic adults with obesity had higher odds of food insecurity (OR, 1.34; 95% CI, 1.11-1.61) compared with those with normal weight.

## Discussion

In this nationally representative sample of US adults, food insecurity increased from 1999 to 2016 in all BMI and WC categories, both sexes, and all racial/ethnic groups. Food insecurity was greater in adults with greater adiposity, including those with generalized and abdominal obesity. Moreover, food insecurity was greater in non-Hispanic Black and Hispanic adults compared with non-Hispanic White adults.

We believe that this cross-sectional study demonstrated notable differences in food insecurity by adiposity in men vs women. Although men had odds of food insecurity similar to those of women, regardless of the level of adiposity, women with greater adiposity were more likely to have food insecurity compared with those with lower adiposity. Furthermore, patterns of food insecurity prevalence by level of adiposity differed between sexes, with the exception of the most recent NHANES cycle of data (2015-2016). Specifically, among women, food insecurity was most prevalent in those with greater adiposity, which was dissimilar from that shown in men from the 1999 to 2014 cycles. These findings are consonant with the preponderance of empirical evidence, demonstrating a robust association between food insecurity and obesity in women.^12,25,26,27^ However, in light of the most recent data, men may be adopting a pattern of food insecurity prevalence by adiposity that is similar to that seen in women.

Trends in food insecurity by adiposity were primarily greater in non-Hispanic Black and Hispanic adults compared with non-Hispanic White adults, but differences were found in patterns of food insecurity prevalence by level of adiposity and race/ethnicity. Non-Hispanic White and Hispanic adults had greater prevalence of food insecurity in those with greater adiposity compared with those with lower adiposity. However, the opposite was shown for non-Hispanic Black adults, with food insecurity being largely greater in those with less adiposity compared with those with greater adiposity. These findings are particularly relevant given a meta-analysis of the published literature regarding the association between food insecurity and obesity in studies that did not include race/ethnicity as a key moderating factor.^12^

The present study points to multiple opportunities to investigate the association between food insecurity and obesity. Such opportunities include biomedical and behavioral research that explores underlying mechanisms and pathways, the potential role of pregnancy in food insecurity and obesity, and racial/ethnic disparities as well as moving beyond cross-sectional analyses to long-term assessments.^28^ Regarding implications for public health and clinical practitioners, this study also highlights a number of important considerations for those aiming to address the growing obesity epidemic among US adults, especially among key population subgroups such as women, racial/ethnic minorities, and adults with low income.^29^ First, screening for food insecurity can aid physicians and other health care practitioners in identifying patients who are vulnerable to being food insecure. Short questionnaires (eg, 2-items) are available that can identify those at risk of experiencing food insecurity.^30,31,32^ In turn, these patients can be connected to support services, such as the charitable food system (eg, food banks, food pantries) and federal nutrition assistance programs. Furthermore, food insecurity screenings can help identify patients who may face barriers (eg, poor nutrition and diet quality, reduced medication adherence) and medical complications (eg, emergency department visits, hospitalizations) that compromise obesity treatment and management. Given the sex- and race/ethnicity-based disparities associated with food insecurity and adiposity found in this study, physicians and other health care practitioners should recognize that prevention and treatment of obesity in specific population subgroups may require multifaceted approaches that address both food insecurity and unhealthy body weight.

### Strengths and Limitations

This study has a number of strengths. First, the NHANES uses the criterion standard assessment of food security status, the 18-item US Household Food Security Survey Module.^2,19^ Second, because the NHANES provides nationally representative data, the results of this study are generalizable to the adult population of the US. Third, because the NHANES is administered continuously, trend analyses by 2-year cycles were possible to better quantify temporal patterns in food insecurity by adiposity. Fourth, we analyzed trends in food insecurity prevalence by relevant population subgroups to explore health disparities by sex and race/ethnicity. Moreover, use of the NHANES allowed for the inclusion of multiple sociodemographic variables, such as educational level and marital status, associated with both food insecurity and adiposity. This study has limitations. First, the use of cross-sectional surveys limited our ability to address directional causality in the association between food insecurity and adiposity. Second, these cross-sectional data also precluded the assessment of long-term changes in food insecurity and adiposity in individuals. This point is especially notable given that food insecurity is considered a cyclic phenomenon with possible episodes of food adequacy and food shortage.^3,4,25^ That is, those with food insecurity may experience periods in which food is more readily available and periods in which food is scarce.^4,33,34^ These episodes of food adequacy and food shortage are unmeasured by the 18-item module, which broadly focuses on the previous 12 months. Third, although this study used the most recent publicly available data (2015-2016), it may not reflect the trends from 2017 onward.

## Conclusions

This cross-sectional study demonstrated that food insecurity in the US has increased from 1999 to 2016, making it a critical public health concern. The continued increase in both food insecurity and obesity warrants further research to identify mechanisms that underlie the association between food insecurity and obesity. Such evidence can be used to develop interventions for reducing food insecurity and obesity as co-occurring conditions. Furthermore, multidisciplinary approaches may be required to address the association between food insecurity and obesity in specific population subgroups, including disparities based on sex and race/ethnicity.

## Notes

Supplement. **eTable 1.** Weighted Sample Characteristics by Food Security Status Among Adults in the US, NHANES 2015-2016 **eFigure 1.** Crude Weighted Trends in Waist Circumference by Sex and Race/Ethnicity, NHANES 1999-2016 **eTable 2.** Crude Weighted Trends in Food Insecurity by Sex and Adiposity Among the US Adult Population, NHANES 1999-2016 **eTable 3.** Crude Weighted Trends in Food Insecurity by Race/Ethnicity and Adiposity Among the US Adult Population, NHANES 1999-2016 **eFigure 2.** Adjusted Odds (95% CI) of Food Insecurity Among Adults in the US, NHANES 1999-2016
