# Supplementary material for: Trends in Adiposity and Food Insecurity Among US Adults
Source: JAMA Netw Open. 2020 Aug 7;3(8):e2012767. doi: 10.1001/jamanetworkopen.2020.12767 (PMC7414390; doi:10.1001/jamanetworkopen.2020.12767)
Supplement: Supplement. — eTable 1. Weighted Sample Characteristics by Food Security Status Among Adults in the US, NHANES 2015-2016 eFigure 1. Crude Weighted Trends in Waist Circumference by Sex and Race/Ethnicity, NHANES 1999-2016 eTable 2. Crude Weighted Trends in Food Insecurity by Sex and Adiposity Among the US Adult Population, NHANES 1999-2016 eTable 3. Crude Weighted Trends in Food Insecurity by Race/Ethnicity and Adiposity Among the US Adult Population, NHANES 1999-2016 eFigure 2. Adjusted Odds (95% CI) of Food Insecurity Among Adults in the US, NHANES 1999-2016 [file jamanetwopen-3-e2012767-s001.pdf]

Supplementary Online Content

Myers CA, Mire EF, Katzmarzyk PT. Trends in adiposity and food insecurity among US adults. *JAMA Netw Open*. 2020;3(8):e2012767.
doi:10.1001/jamanetworkopen.2020.12767

eTable 1. Weighted Sample Characteristics by Food Security Status Among Adults in the US, NHANES 2015-2016

eFigure 1. Crude Weighted Trends in Waist Circumference by Sex and Race/Ethnicity, NHANES 1999-2016

eTable 2. Crude Weighted Trends in Food Insecurity by Sex and Adiposity Among the US Adult Population, NHANES 1999-2016

eTable 3. Crude Weighted Trends in Food Insecurity by Race/Ethnicity and Adiposity Among the US Adult Population, NHANES 1999-2016

eFigure 2. Adjusted Odds (95% CI) of Food Insecurity Among Adults in the US, NHANES 1999-2016

This supplementary material has been provided by the authors to give readers additional information about their work.

**eTable 1.** Weighted Sample Characteristics by Food Security Status Among Adults in the US, NHANES 2015-2016[a]			
	**Food Secure**	**Food Insecure**	***p-value***[c]
Unweighted sample	3,978	1,331	
Weighted sample[b]	186,788,670	41,645,622	
Body mass index (BMI)			<0.001
<25 kg/m^2	29.3 (260.-32.7)	23.4 (18.5-28.4)	
25 to 29.9 kg/m^2	32.7 (31.1-34.3)	27.0 (23.2-30.9)	
≥30 kg/m^2	37.9 (34.3-41.5)	49.5 (45.2-53.9)	
Waist circumference (WC)			0.006
Less than High-Risk	44.5 (39.8-49.1)	38.5 (34.1-42.8)	
High-Risk	55.5 (50.9-60.2)	61.5 (57.2-65.9)	
Sex			0.006
Female	51.5 (50.0-53.1)	54.9 (52.6-57.2)	
Male	48.5 (46.9-50.0)	45.1 (42.8-47.4)	
Race/ethnicity			<0.001
Non-Hispanic White	68.3 (60.4-76.3)	45.9 (35.5-56.3)	
Non-Hispanic African American	9.9 (5.5-14.2)	18.2 (11.3-25.0)	
Hispanic	11.9 (6.9-16.9)	28.7 (21.0-36.5)	
Other	9.9 (6.9-12.9)	7.2 (4.8-9.5)	
Age			<0.001
20-39	34.0 (31.0-37.0)	44.7 (40.2-49.1)	
40-64	43.9 (41.5-46.4)	42.6 (39.9-45.3)	
65 and older	22.0 (19.3-24.8)	12.7 (9.0-16.5)	
Education			<0.001
High school or less	31.1 (26.2-35.9)	53.2 (47.9-58.5)	
Some college or more	68.9 (64.1-73.8)	46.8 (41.5-52.1)	
Poverty to Income Ratio (family)			<0.001
≤130%	19.3 (15.8-22.7)	53.9 (49.4-58.5)	
>130%	80.7 (77.3-84.2)	46.1 (41.5-50.6)	
Marital Status			<0.001
Married	57.6 (53.8-61.5)	40.2 (34.7-45.6)	
Not married	42.4 (38.5-46.2)	59.8 (54.4-65.3)	

[a] Data are presented as percentage % (95% CI).
[b] Data are weighted to be nationally representative.

©2020 Myers CA et al. *JAMA Network Open*

[c] Chi-square tests to determine statistically significant differences between food security status groups.
[d] Other includes race/ethnicity other than Non-Hispanic White, Non-Hispanic African American, or Hispanic, including multiracial.

eFigure 1. Crude Weighted Trends in Waist Circumference by Sex and Race/Ethnicity, NHANES 1999-2016[a]

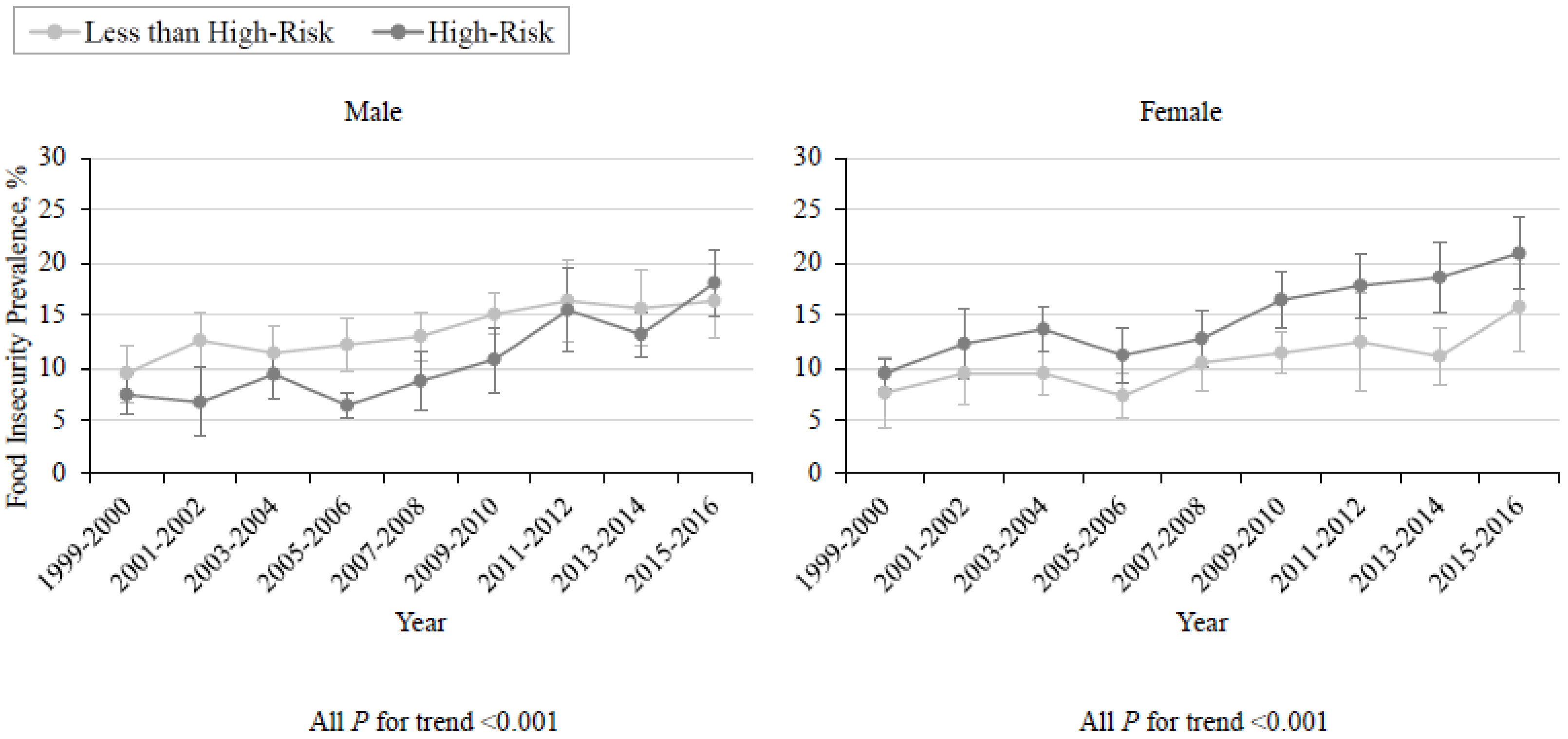

©2020 Myers CA et al. *JAMA Network Open*

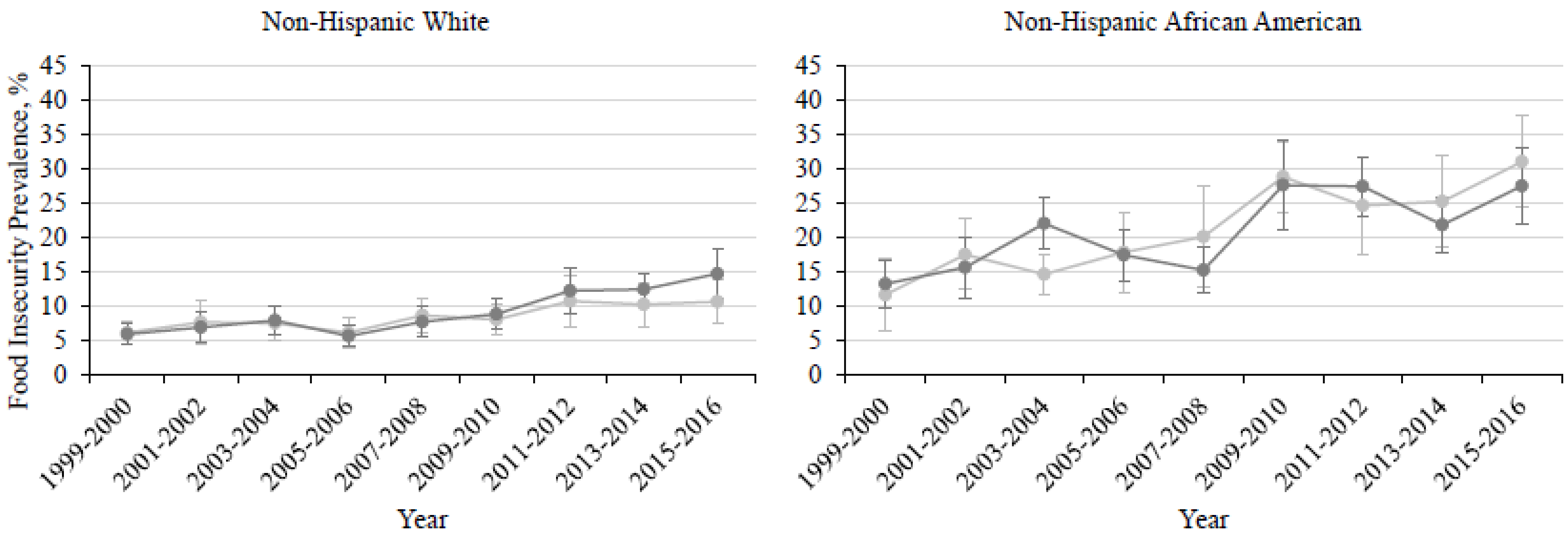

Less than High-Risk (*P* for trend =0.003)
High-Risk (*P* for trend <0.001)

All *P* for trend <0.001

©2020 Myers CA et al. *JAMA Network Open*

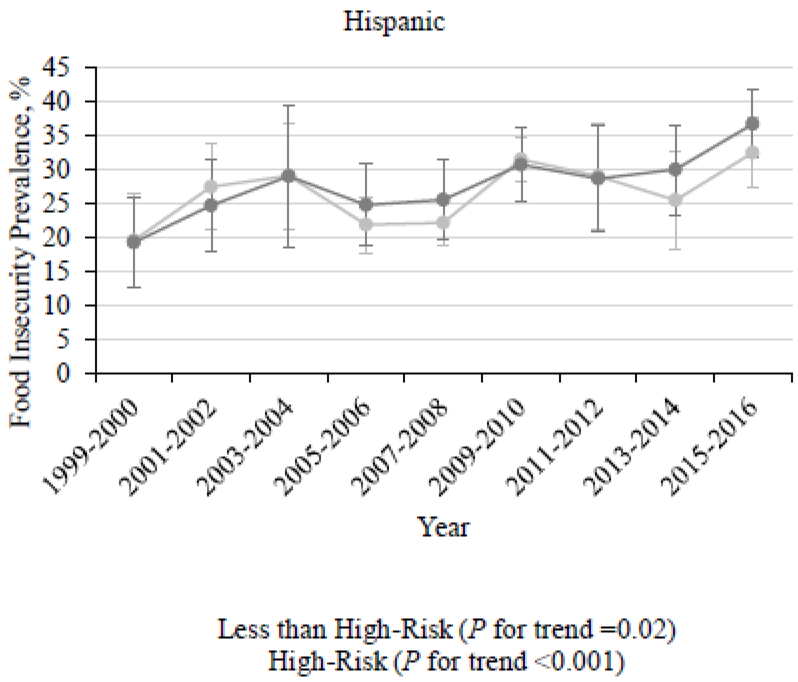

[a] Data weighted to be nationally representative. Error bars indicate 95% CIs.

©2020 Myers CA et al. *JAMA Network Open*

**eTable 2.** Crude Weighted Trends in Food Insecurity by Sex and Adiposity Among the US Adult Population, NHANES 1999-2016[a,b]										
	**1999-2000**	**2001-2002**	**2003-2004**	**2005-2006**	**2007-2008**	**2009-2010**	**2011-2012**	**2013-2014**	**2015-2016**	***P* for Trend**[c]
Female										
**BMI**										
<25 kg/m^2	7.3 (4.7 – 9.9)	8.2 (5.8 – 10.5)	9.2 (7.0 – 11.3)	7.8 (5.1 – 10.4)	8.8 (6.2 – 11.5)	10.8 (8.5 – 13.1)	12.3 (8.2 – 16.5)	10.6 (7.3 – 13.8)	14.7 (9.9-19.4)	<0.001
25 to 29.9 kg/m^2	7.0 (4.4 – 9.7)	10.2 (6.3 - 14.1)	11.1 (7.6 – 14.7)	8.6 (5.8 – 11.5)	10.8 (8.4 – 13.3)	12.6 (9.7 – 15.5)	13.2 (9.2 – 17.2)	13.9 (9.6 – 18.2)	16.1 (11.9-20.3)	<0.001
≥30 kg/m^2	11.8 (9.6 – 14.0)	14.5 (10.9 – 18.0)	16.0 (13.3 – 18.6)	12.2 (8.8– 15.6)	15.6 (11.8 – 19.4)	19.6 (15.5 – 23.6)	21.3 (17.2 – 25.4)	21.4 (17.4 – 25.4)	24.6 (20.8-28.4)	<0.001
**WC**										
Less than High-Risk	7.7 (4.4 – 11.0)	9.5 (6.6 – 12.3)	9.5 (7.4 – 11.5)	7.4 (5.2 – 9.6)	10.5 (7.9 – 13.1)	11.4 (9.5 – 13.4)	12.5 (7.9 – 17.0)	11.1 (8.3 – 13.8)	15.8 (11.6-20.0)	<0.001
High-Risk	9.5 (8.1 – 10.9)	12.3 (9.0 – 15.7)	13.7 (11.6 – 15.9)	11.2 (8.6 – 13.8)	12.8 (10.1 – 15.4)	16.5 (13.9 – 19.2)	17.8 (14.8 – 20.8)	18.6 (15.3 – 21.9)	20.9 (17.5-24.4)	<0.0001
Male										
**BMI**										
<25 kg/m^2	10.4 (6.5 – 14.4)	14.5 (9.9 – 19.1)	12.2 (8.1 – 16.3)	13.1 (9.3 – 16.9)	14.2 (11.3 – 17.2)	14.9 (12.4 – 17.5)	16.1 (10.6 – 21.6)	18.8 (13.5 – 24.1)	15.6 (11.3-20.4)	0.008
25 to 29.9 kg/m^2	7.5 (5.2 – 9.8)	9.3 (7.4 - 11.3)	8.8 (6.1 – 11.5)	9.3 (7.2 – 11.5)	10.1 (7.6 – 12.6)	13.7 (10.7 – 16.6)	14.1 (9.9 – 18.2)	11.7 (8.6 – 14.8)	15.1 (10.8-19.5)	<0.001
≥30 kg/m^2	8.6 (5.7 – 11.5)	7.5 (3.9 – 11.2)	11.1 (8.9 – 13.3)	7.2 (5.3 – 9.0)	10.5 (7.0 – 14.0)	11.5 (8.4 – 14.6)	17.8 (13.6 – 22.0)	14.7 (12.5 – 16.9)	20.2 (16.5-24.0)	<0.001
**WC**										
Less than High-Risk	9.5 (6.8 – 12.2)	12.6 (10.0 – 15.2)	11.4 (8.9 – 13.9)	12.2 (9.6 – 14.7)	13.0 (10.7 – 15.3)	15.1 (13.1 – 17.0)	16.4 (12.5 – 20.2)	15.7 (12.0 – 19.3)	16.4 (12.9-20.0)	<0.001
High-Risk	7.5 (5.5 – 9.4)	6.8 (3.5 – 10.0)	9.4 (7.2 – 11.7)	6.5 (5.3 – 7.7)	8.8 (6.0 – 11.6)	10.8 (7.8 – 13.9)	15.5 (11.5 – 19.5)	13.2 (11.1 – 15.4)	18.1 (14.9-21.3)	<0.001

[a] Data are presented as percentage % (95% CI) for each survey cycle.
[b] Data are weighted to be nationally representative.
[c] The estimate β (95% CI) and P for trend were calculated using logistic regression that included the NHANES 2-year cycle as a continuous variable.

eTable 3. Crude Weighted Trends in Food Insecurity by Race/Ethnicity and Adiposity Among the US Adult Population, NHANES 1999-2016[a,b]

	1999-2000	2001-2002	2003-2004	2005-2006	2007-2008	2009-2010	2011-2012	2013-2014	2015-2016	*P* for Trend
Non-Hispanic White										
**BMI**										
<25 kg/m^2	6.8 (3.6 – 10.0)	8.1 (4.5 – 11.7)	8.5 (5.1 – 11.9)	6.4 (4.1 – 8.8)	8.6 (5.7 – 11.5)	8.5 (6.2 – 10.9)	11.6 (6.7 – 16.5)	11.2 (7.0 – 15.4)	11.7 (7.4-15.9)	0.006
25 to 29.9 kg/m^2	3.9 (1.6 – 6.2)	5.3 (3.6 – 7.0)	5.7 (3.7 – 7.7)	5.3 (3.5– 7.1)	6.9 (5.1 – 8.8)	7.5 (5.5 – 9.5)	8.7 (5.7 – 11.8)	8.6 (5.5 – 11.7)	9.6 (5.8-13.4)	<0.001
≥30 kg/m^2	7.6 (4.9 – 10.3)	8.0 (4.8 – 11.2)	9.2 (6.9 – 11.4)	5.5 (3.4 – 7.7)	8.9 (5.8 – 12.0)	9.1 (6.6 – 11.5)	14.3 (9.8 – 18.8)	14.4 (11.1 – 17.7)	17.1 (13.0-21.1)	<0.001
**WC**										
Less than High-Risk	6.1 (4.3 – 7.5)	7.6 (4.4 – 10.8)	7.5 (5.0 – 9.9)	6.1 (3.8 – 8.4)	8.6 (6.2 – 11.0)	8.0 (5.7 – 10.2)	10.7 (6.9 – 14.4)	10.2 (7.0 – 13.4)	10.6 (7.5-13.8)	0.003
High-Risk	5.9 (4.3 – 7.5)	6.9 (4.6 – 9.1)	7.9 (5.7 – 10.1)	5.6 (4.0 – 7.1)	7.7 (5.5 – 10.0)	8.8 (6.5 – 11.1)	12.2 (8.8 – 15.5)	12.4 (10.0 – 14.8)	14.7 (11.0-18.4)	<0.001
Non-Hispanic African American **BMI**										
<25 kg/m^2	13.3 (6.6 – 20.1)	18.0 (9.6 – 26.5)	13.5 (7.6 – 19.5)	20.8 (13.5 – 28.0)	21.7 (13.8 – 29.7)	31.4 (26.0 – 36.8)	25.2 (14.7 – 35.6)	29.6 (21.1 – 38.1)	34.0 (24.1-43.9)	<0.001
25 to 29.9 kg/m^2	10.3 (5.0 – 15.6)	15.8 (11.0 - 20.7)	16.9 (8.4 – 25.5)	15.2 (10.7 – 19.7)	13.6 (7.6 – 19.7)	25.5 (16.6 – 34.3)	23.9 (19.1 – 28.7)	22.9 (17.9 – 27.9)	27.0 (22.6-31.5)	<0.001
≥30 kg/m^2	13.0 (9.1 – 16.9)	16.6 (11.3 – 21.9)	21.5 (17.0 – 25.9)	17.5 (13.1 – 21.9)	17.8 (14.0– 21.6)	28.0 (21.0 – 35.0)	28.3 (22.4 – 34.1)	20.6 (16.6 – 24.7)	28.3 (22.6-34.1)	<0.001
**WC**										
Less than High-Risk	11.6 (6.3 – 16.9)	17.5 (12.3 – 22.6)	14.6 (11.7 – 17.5)	17.8 (12.1 – 23.6)	20.1 (12.8 – 27.5)	28.8 (23.6 – 34.0)	24.6 (17.5 – 31.7)	25.2 (18.6 – 31.8)	31.0 (24.3-37.6)	<0.001
High-Risk	13.2 (9.6 – 16.7)	15.6 (11.1 – 20.0)	22.0 (18.3 – 25.7)	17.4 (13.6 – 21.2)	15.2 (11.9 – 18.5)	27.6 (21.0 – 34.1)	27.4 (23.2 – 31.7)	21.8 (17.7 – 25.9)	27.5 (21.9-33.2)	<0.001
Hispanic										
**BMI**										
<25 kg/m^2	18.4 (9.1 – 27.7)	26.2 (18.2 – 34.2)	30.6 (19.8 – 41.4)	18.4 (12.1 – 24.6)	20.2 (12.8 – 27.6)	25.6 (21.1 – 30.4)	23.8 (16.6 – 30.9)	25.2 (16.7 – 33.6)	28.1 (23.1-33.0)	0.34
25 to 29.9 kg/m^2	20.1 (13.8 – 26.3)	29.4 (21.3 – 37.5)	29.2 (19.1 – 39.3)	23.6 (18.5 – 28.7)	23.6 (19.0 – 28.2)	30.9 (24.7 – 37.2)	29.4 (20.3– 38.5)	25.0 (19.9 – 30.1)	34.7 (27.0-42.4)	0.06
≥30 kg/m^2	19.1 (12.0 – 26.1)	23.1 (14.9 – 31.2)	27.6 (15.5 – 39.7)	26.9 (19.9 – 33.8)	26.8 (19.6 – 34.0)	34.2 (28.4 – 39.9)	30.8 (22.5 – 39.1)	31.0 (23.4 – 38.6)	37.6 (33.7-41.5)	<0.001
**WC**										
Less than High-Risk	19.6 (12.8 – 26.4)	27.5 (21.2 – 33.7)	29.1 (21.3 – 36.9)	21.9 (17.8 – 26.1)	22.2 (18.9 – 25.4)	31.5 (28.2 – 34.7)	29.0 (21.1 – 36.9)	25.5 (18.4 – 32.6)	32.5 (27.3-37.7)	0.02
High-Risk	19.3	24.7	29.0	24.8	25.6	30.7	28.7	30.0	36.7	<0.001

	(12.7 – 25.9)	(18.1 – 31.4)	(18.4 – 39.4)	(18.7 – 30.8)	(19.8 – 31.5)	(25.3 – 36.1)	(21.1 – 36.4)	(23.4 – 36.6)	(31.7-41.7)	

[a] Data are presented as percentage % (95% CI) for each survey cycle.
[b] Data are weighted to be nationally representative.
[c] The estimate β (95% CI) and P for trend were calculated using logistic regression that included the NHANES 2-year cycle as a continuous variable.

eFigure 2. Adjusted Odds (95% CI) of Food Insecurity Among Adults in the US, NHANES 1999-2016[a]

A. Male

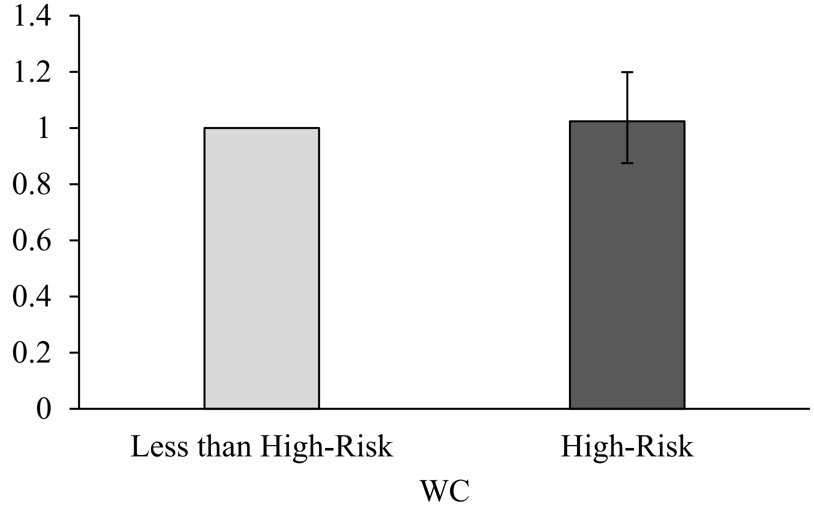

©2020 Myers CA et al. *JAMA Network Open*

B. Female

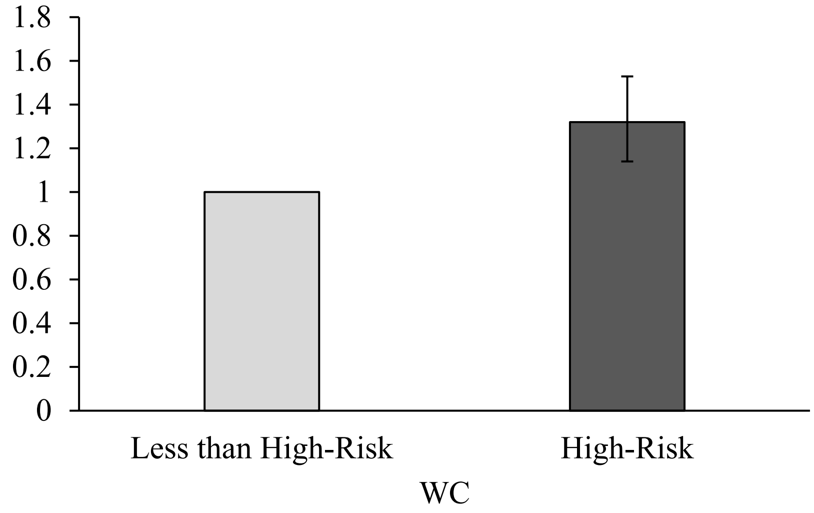

C. Non-Hispanic White

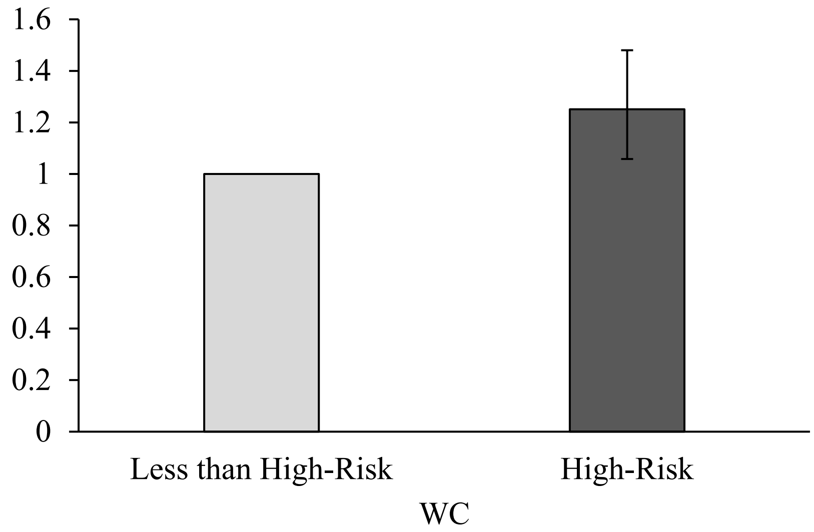

©2020 Myers CA et al. *JAMA Network Open*

D. Non-Hispanic African American

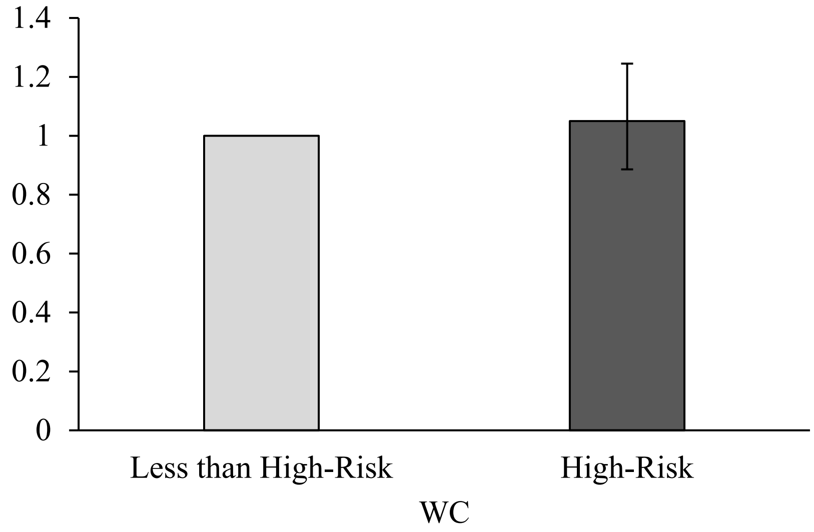

E. Hispanic

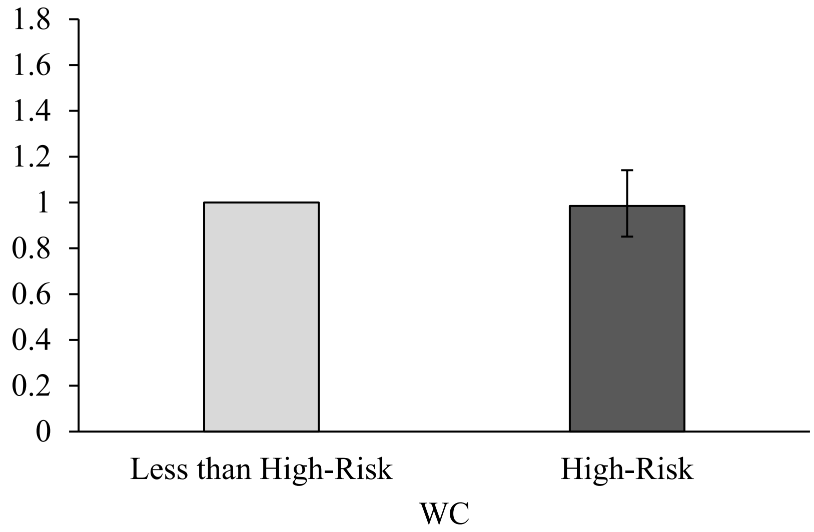

[a]Data weighted to be nationally representative. Error bars indicate 95% CI.

©2020 Myers CA et al. *JAMA Network Open*
